# Isolation and characterization of myogenic precursor cells from human cremaster muscle

**DOI:** 10.1038/s41598-019-40042-6

**Published:** 2019-03-05

**Authors:** Neia Naldaiz-Gastesi, María Goicoechea, Isabel M-ª Aragón, Virginia Pérez-López, Sandra Fuertes-Alvarez, Bernardo Herrera-Imbroda, Adolfo López de Munain, Resi de Luna-Diaz, Pedro M. Baptista, M. Alejandro Fernández, María Fernanda Lara, Ander Izeta

**Affiliations:** 1 Tissue Engineering group, Instituto Biodonostia, San Sebastian, Spain; 2 Neuromuscular diseases group, Instituto Biodonostia, San Sebastian, Spain; 3 Centro de Investigación Biomédica en Red de Enfermedades Neurodegenerativas (CIBERNED), Instituto de Salud Carlos III, Madrid, Spain; 4 CNIO-IBIMA Genitourinary Cancer Research Unit, Institute of Biomedical Research in Málaga (IBIMA), Málaga, Spain; 5 Urology Department, Hospital Universitario Virgen de la Victoria, Málaga, Spain; 6 Department of Neurosciences, Faculty of Medicine and Dentistry, UPV-EHU, San Sebastian, Spain; 7 Department of Neurology, Hospital Universitario Donostia, San Sebastian, Spain; 8 Department of Surgery, Hospital Universitario Virgen de la Victoria, Málaga, Spain; 9 Health Research Institute of Aragón (IIS Aragón), Zaragoza, Spain; 10 Centro de Investigación Biomédica en Red de Enfermedades Hepáticas y Digestivas (CIBEREHD), Instituto de Salud Carlos III, Madrid, Spain; 11 Health Research Institute of Jiménez Díaz Foundation (IIS FJD), Madrid, Spain; 12 Biomedical and Aerospace Engineering Department, University Carlos III of Madrid, Madrid, Spain; 13 Digital industry, Product design, IDONIAL Technology Center, Gijón, Spain; 14 Department of Biomedical Engineering and Science, School of Engineering, Tecnun-University of Navarra, San Sebastian, Spain; 15 Present Address: Viralgen Vector Core, San Sebastian, Spain

## Abstract

Human myogenic precursor cells have been isolated and expanded from a number of skeletal muscles, but alternative donor biopsy sites must be sought after in diseases where muscle damage is widespread. Biopsy sites must be relatively accessible, and the biopsied muscle dispensable. Here, we aimed to histologically characterize the cremaster muscle with regard number of satellite cells and regenerative fibres, and to isolate and characterize human cremaster muscle-derived stem/precursor cells in adult male donors with the objective of characterizing this muscle as a novel source of myogenic precursor cells. Cremaster muscle biopsies (or adjacent non-muscle tissue for negative controls; N = 19) were taken from male patients undergoing routine surgery for urogenital pathology. Myosphere cultures were derived and tested for their *in vitro* and *in vivo* myogenic differentiation and muscle regeneration capacities. Cremaster-derived myogenic precursor cells were maintained by myosphere culture and efficiently differentiated to myotubes in adhesion culture. Upon transplantation to an immunocompromised mouse model of cardiotoxin-induced acute muscle damage, human cremaster-derived myogenic precursor cells survived to the transplants and contributed to muscle regeneration. These precursors are a good candidate for cell therapy approaches of skeletal muscle. Due to their location and developmental origin, we propose that they might be best suited for regeneration of the rhabdosphincter in patients undergoing stress urinary incontinence after radical prostatectomy.

## Introduction

In striated muscle, adult myogenic stem cells are known as satellite cells, due to their superficial position on muscle fibres^1^. The myogenic process is a multifaceted transition between precursor states (quiescence, activation, proliferation and differentiation) that precede fusion of the myoblasts to regenerative muscle fibres^2^. Besides, satellite cells reside in a complex niche, which includes other precursors such as fibro-adipogenic precursor cells (FAPs) that modulate the regenerative response^3^, along with signals arising from nerve and capillary terminals and other interstitial cells. For cell-based therapeutic purposes, it would thus be desirable to obtain and characterize the diverse types of human muscle precursor cells from an accessible source.

Most protocols of human satellite cell isolation rely on the purification of cell subpopulations by flow cytometry or magnetic separation of muscle-derived cell suspensions through differential expression of membrane markers^4–21^. Despite the important recent advances in the purification and characterization of human satellite cells, they are still isolated in small numbers out of muscle biopsies of a limited size (typically of 50–100 mg; there are between 500–1,000 satellite cells per mm^3^ ^20^), and the stem cells present restricted expansion capacities *in vitro*^22,23^. For these reasons, myoblasts (a heterogeneous mixture of semi-purified CD56+ cells^24,25^) have been mostly used in clinical trials to date, since they expand well *in vitro*^26,27^. However, the results of expanded myoblast cell infusion in a plethora of clinical trials targeting muscle regeneration have been disappointing^28–32^. Some authors argue that the large expansion rates of myoblasts have brought up excessive differentiation in culture and hence low survival, migration and fusogenic capacities when cells are transplanted *in vivo*^33–35^. Of note, more experiments must be done with human myoblasts to ensure that this is also the case in humans^36^.

A still relatively unexplored possibility is the growth of human muscle precursor cells in three-dimensional myosphere cultures^37^. In mice, myospheres represent a mixture of ITGA7+ Myf5+ MyoD+Pax7+ myogenic (possibly satellite cells) precursor cells and non-myogenic (possibly FAPs) precursors characterized as PDGFRα+Sca−1+^38,39^. Since both precursor cell populations are required for muscular regeneration, myosphere cultures would have the advantage of providing two precursor cell types instead of one, when compared to alternative satellite cell isolation strategies. Human myosphere cultures present at least a CD31−CD34−CD45−CD56−CD117−CD29+CD73+CD90+CD105+ “mesenchymal” stem cell population, which possibly is non-myogenic^40^ and could be equivalent to FAPs, and a CD34−CD45−PAX7+CD56+ALDH1+ myogenic precursor cell population that possibly corresponds to satellite cells^41^.

In this article, we propose human cremaster muscle as a convenient source of muscle-derived stem/precursor cells in male donors. This is a striated muscle not inserted through a tendon, and which also contains a variable number of smooth muscle fibres^42^. Predominantly, it is composed of type I (slow) fibres, although it also contains some of IIB (very fast) type. The function of this muscle in the adult is to contribute to the thermoregulation and protection of testicles, and we postulate it should be classified as an evolutionary remnant of mammalian *Panniculus carnosus* muscle^43^. Thanks to the cremasteric reflex, its electrophysiological properties are well known. The muscle is densely innervated and presents numerous motor endplates, which may be the reason underlying its abundant spontaneous discharges^42^. In children, no sexual dimorphism was observed in cremaster muscle except for a larger diameter of fibres in males, as it is commonly observed in most muscular groups^44^. In embryonic development, cremaster muscle derives from the gubernaculum, independent of the internal oblique muscle of the abdomen, and it performs a key function in testicular descent^45–47^. However, some authors propose that striated cremaster fibres transdifferentiate from smooth muscle instead^48^, as it may happen in other muscles of the genitourinary tract, such as the rhabdosphincter^49^.

Since alternative donor biopsy sites must be identified in diseases where muscle affection is widespread, we here aimed to histologically characterize the cremaster muscle with regard number of satellite cells and regenerative fibres, and to isolate and characterize human cremaster muscle-derived stem/precursor cells in adult male donors to evaluate this muscle as a novel source of myogenic precursor cells.

## Results

### Histological characterization of human cremaster muscle

**Figure 1:**
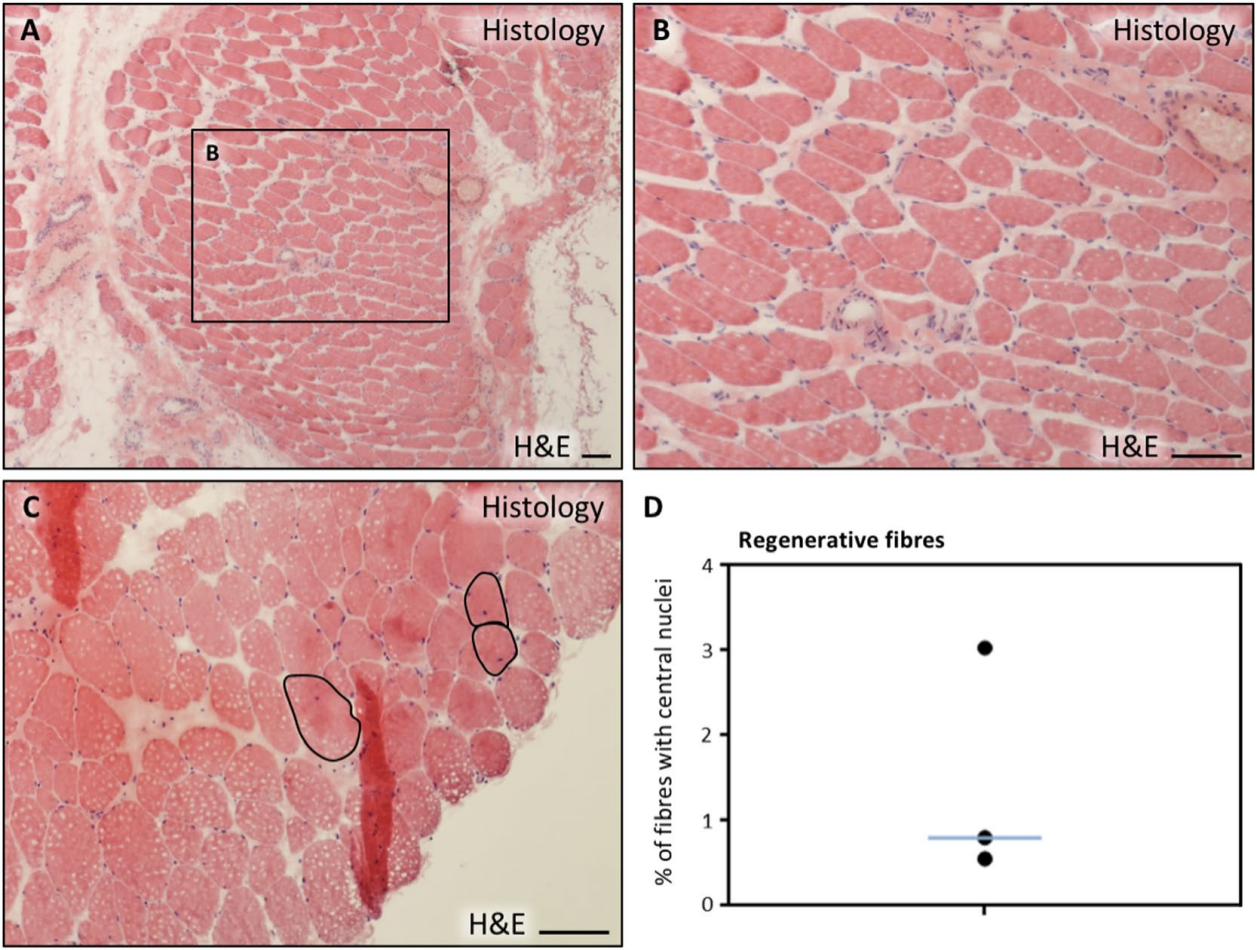
Histological characterization of human cremaster muscle. (**A**–**C**) Histological section of cremaster muscle stained with H&E where detailed fibre morphology can be seen (**A**,**B**) and the presence of regenerative fibres with central nuclei is highlighted (**C**,**D**) Percentage of centrally nucleated fibres as quantified in three independent biopsies. The blue line represents the median value of the data. Scale bars, 100 μm.

**Figure 2:**
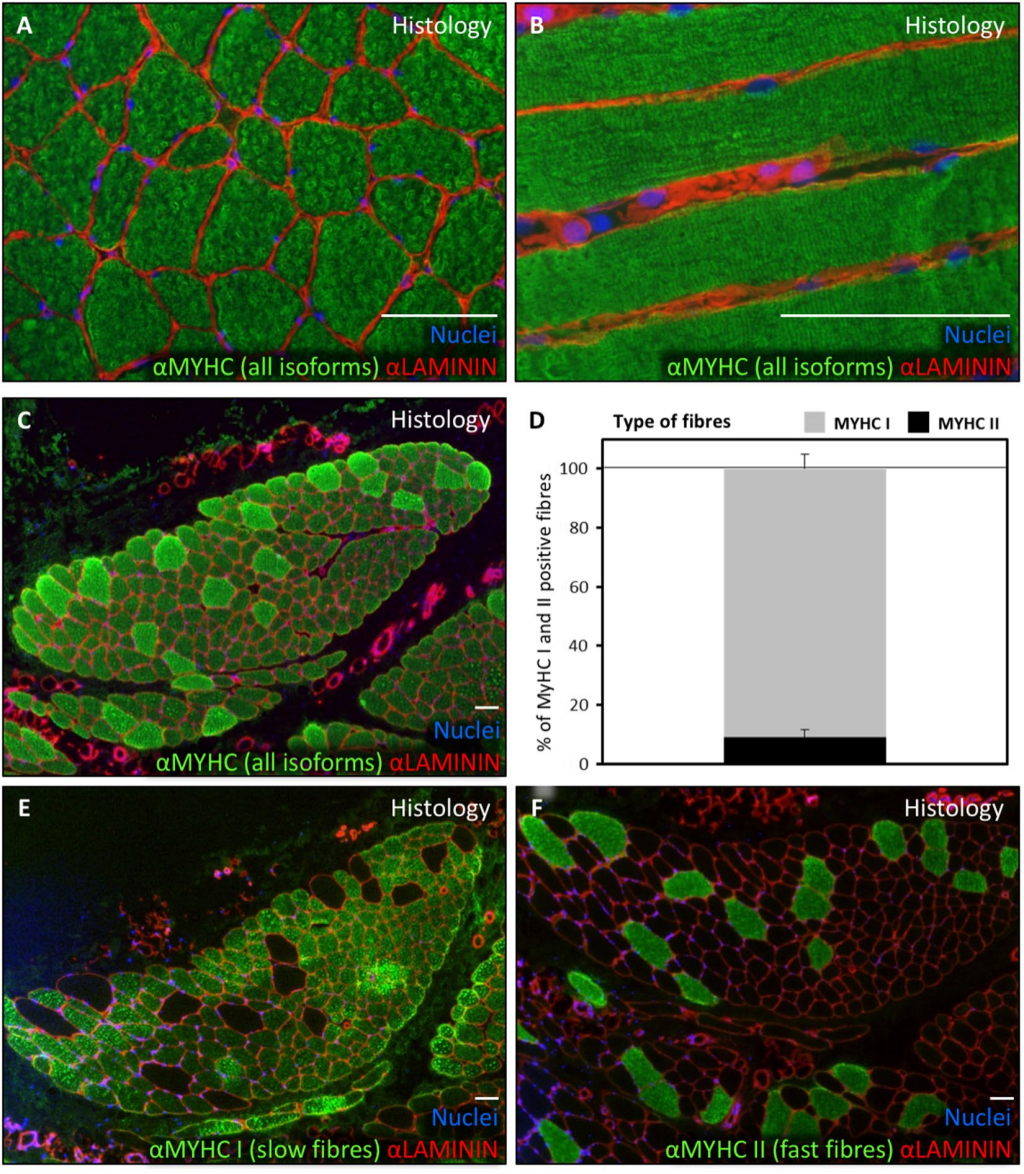
Predominance of slow type fibres in human cremaster muscle. (**A**,**B**) Muscle sections were analysed by immunofluorescence to show MYHC expression in the fibres (all isoforms, green) and LAMININ expression surrounding the fibres (red). (**C**,**D**) Fibre type predominance study through myosin heavy chain isoform expression analysis by immunofluorescence (**C**,**E**,**F**) and resulting distinct fibre quantification graph showing median and standard deviation obtained from three independent biopsies (**D**). In A, B, C, E and F panels LAMININ is shown in red and nuclei are counterstained with Hoechst 33258 (blue). Scale bars, 100 μm.

**Figure 3:**
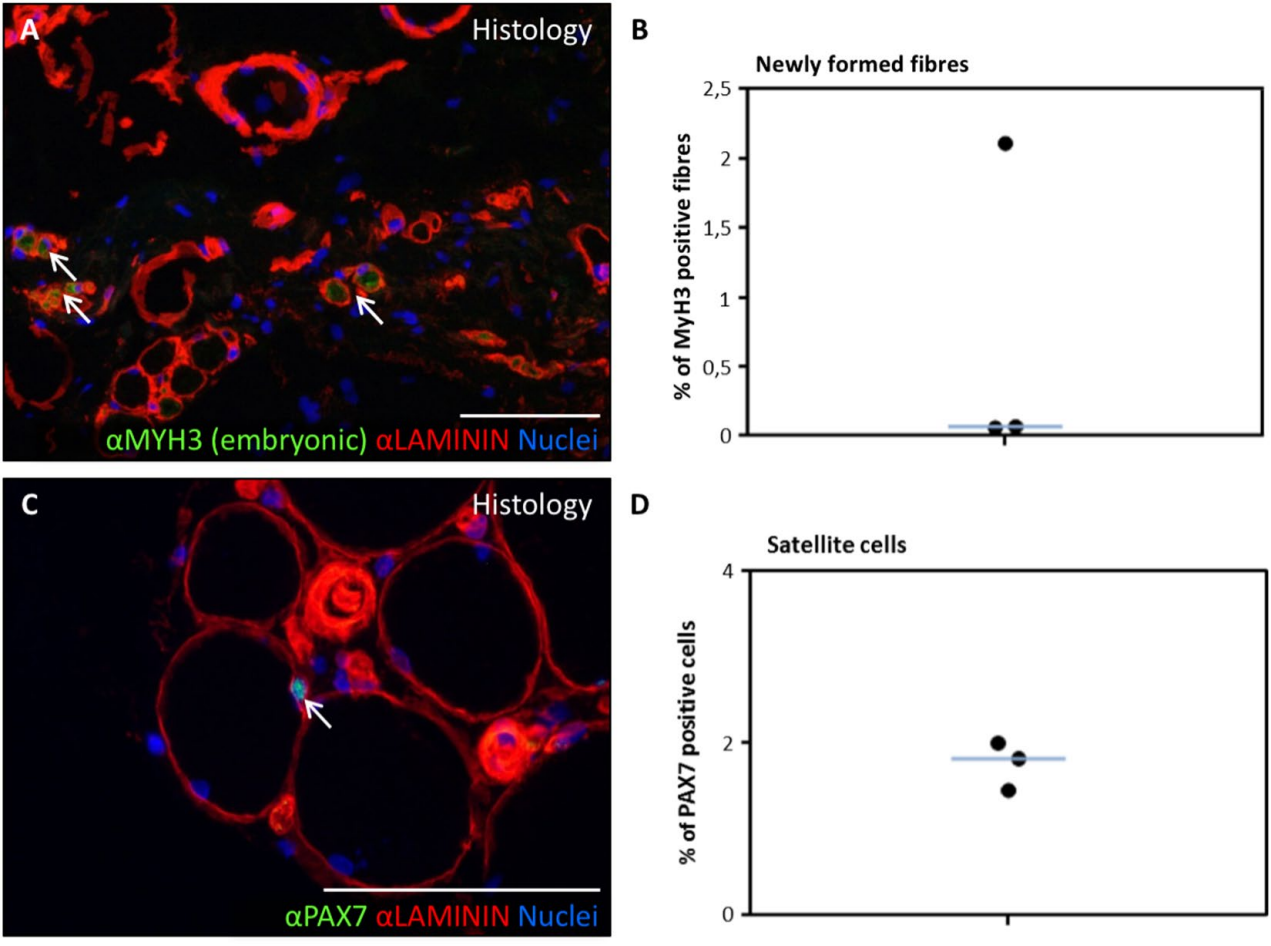
Satellite cells and regenerative fibres in human cremaster muscle. (**A**,**B**) Percentage of MYH3 (embryonic myosin isoform)-expressing fibres detected by immunofluorescence (green), surrounded by LAMININ (red). Arrows in (**A**) show regenerative fibres and the graph (**B**) shows regenerative fibres as a percentage of total fibres, as quantified in three independent biopsies. The blue line represents the median value of the data. (**C**,**D**) Immunofluorescence detection of PAX7+ satellite cells (green) located in their niche between two LAMININ positive layers (red). The arrow in (**C**) points to a satellite cell. (**D**) Percentage of PAX7 positive nuclei as quantified in three independent biopsies. The blue line represents the median value of the data. Nuclei were counterstained with Hoechst 33258 (blue). Scale bars, 100 μm.

The cremaster muscle is surgically accessible in the context of male patients undergoing routine surgery for urogenital pathology (mainly hydrocele and varicocele). Histological characterization (haematoxylin and eosin stain) of cremaster muscle biopsies of these patients (Table 1) showed the presence of a discrete percentage (0.5–3%) of centrally nucleated, regenerative striated fibres as well as some interspersed smooth muscle fibres (Fig. 1), as expected. By immunofluorescence, striated fibre sarcomeres were clearly delineated by myosin heavy chain (MYHC all fibres) antibody staining, and muscle fibres were surrounded by LAMININ positive basal membrane (Fig. 2A,B). Predominance of type I (slow) fibres and the presence of fewer number of type II (fast) fibres was corroborated by the expression of specific MYHC I and MYHC II isoforms, respectively (Fig. 2C–F). The existence of newly formed fibres was confirmed by expression of the embryonic isoform of MYHC, MYH3 (Fig. 3A,B, arrows). To quantify the number of satellite cells *in situ*, the number of PAX7+ nuclei that were surrounded by LAMININ+ basal membrane was determined (Fig. 3C,D, arrow). A proportion of 1.8 ± 0.3% satellite cells were calculated.

**Table 1:** Characteristics of biopsy donors in this study.

Donor #	Sex	Age (y)	Department	Pathology	Tissue^a^
1	Male	35	Urology	Spermatic cord cyst	Non-muscle tissue
2	Male	26	Urology	Varicocele	Cremaster
3	Male	22	Urology	Varicocele	Cremaster
4	Male	64	Urology	Hydrocele	Non-muscle tissue
5	Male	48	Urology	Hydrocele	Non-muscle tissue
6	Male	50	Urology	Hydrocele	Cremaster
7	Male	31	Urology	Varicocele	Cremaster
8	Male	17	Urology	Varicocele	Cremaster
9	Male	20	Urology	Hydrocele	Cremaster
10	Male	35	General surgery	Inguinal hernia	Cremaster
11	Male	51	General surgery	Inguinal hernia	Cremaster
12	Male	71	General surgery	Inguinal hernia	Cremaster
13	Male	29	General surgery	Inguinal hernia	Cremaster
14	Male	50	General surgery	Inguinal hernia	Cremaster
15	Male	70	General surgery	Inguinal hernia	Cremaster
16	Male	45	General surgery	Inguinal hernia	Cremaster
17	Male	86	General surgery	Inguinal hernia	Cremaster
18	Male	74	General surgery	Inguinal hernia	Cremaster
19	Male	41	General surgery	Inguinal hernia	Cremaster

^a^Some biopsies were taken for negative control and included non-muscle tissue adjacent to the Cremaster muscle.

### *In vitro* myotube formation from human cremaster muscle-derived cells

**Figure 4:**
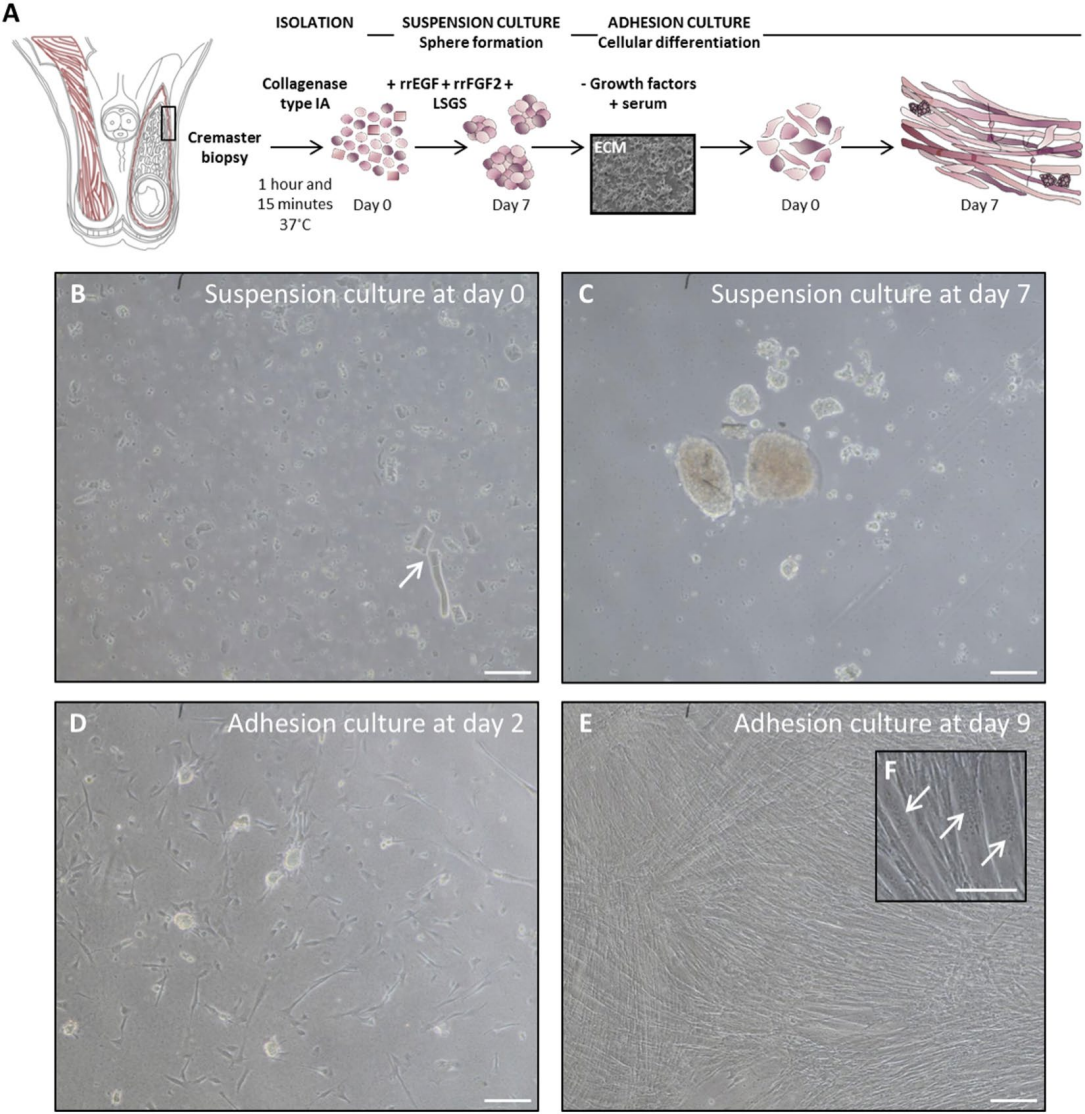
*In vitro* isolation, expansion and differentiation of human cremaster muscle-derived myogenic precursor cells. (**A**) Schematic representation of the male reproductive system anatomy, showing cremaster muscle (red) and the biopsy sample zone is highlighted by a rectangle. Outline of the myosphere suspension culture and myotube differentiation (adhesion culture) steps. (**B**,**C**) Optical microscope images of the suspension culture showing cells and tissue fragments (arrow) at day 0 (**B**) and spheres at day 7 (**C**). (**D**–**F**) Optical microscope images of the adhesion culture at day 2 (**D**) and day 9 (**E**,**F**), where multinucleated myotubes (arrows) become predominant. Scale bars, 100 μm.

To evaluate *in vitro* myogenic potential of human cremaster muscle-derived cells, a protocol previously used in mouse cultures^50^ was adapted to human biopsies (Fig. 4A). At day 0 (d0), suspension cultures presented abundant cellular debris and dead cells as well as unicellular suspensions and muscle tissue remnants (Fig. 4B, arrow). After 7 days of myosphere culture, cells formed spheres of variable size (Fig. 4C). These spheres were then put into differentiation culture^51^. The cells adhered to the substrate and started forming multinucleated myotubes by d2 (Fig. 4D) and, by d9, myotubes occupied most of the culture surface (Fig. 4E,F, arrows). These results suggested that human cremaster muscle-derived cells adopted a myogenic commitment *in vitro* in response to appropriate cues.

**Figure 5:**
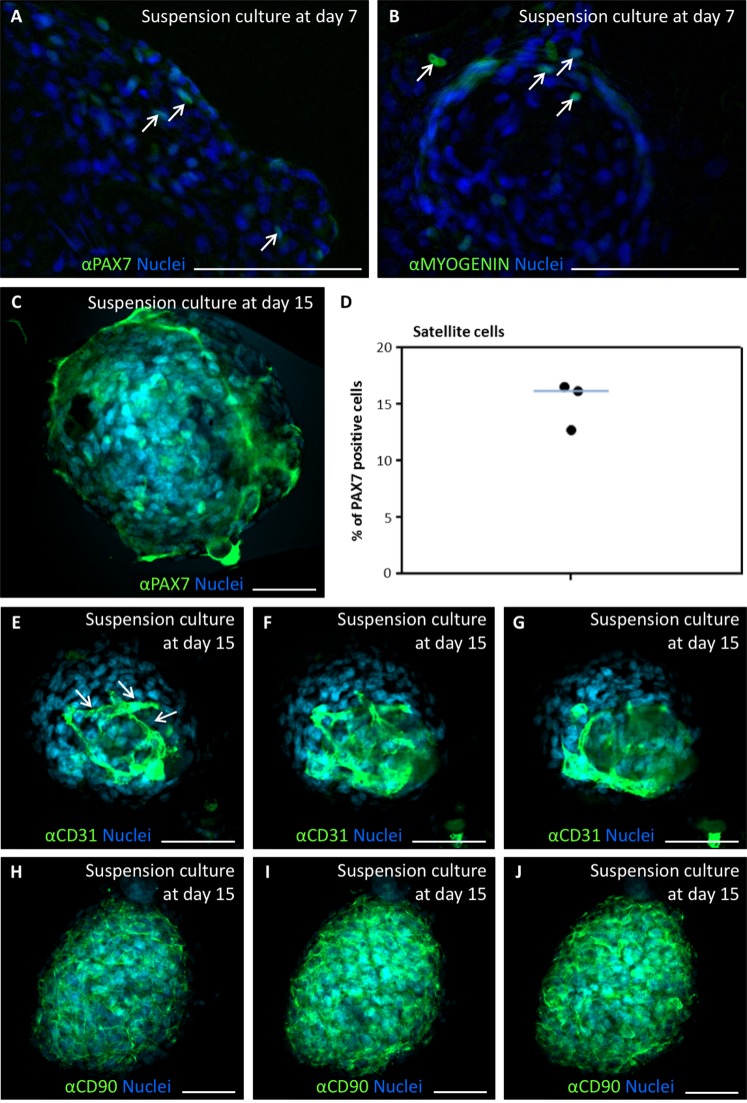
Expression of myogenic, endothelial and mesenchymal markers during *in vitro* expansion of human cremaster muscle-derived myogenic precursor cells. (**A**–**C**) Immunofluorescence analysis of myogenic protein expression in d7 (**A**,**B**) and d15-cultured myospheres (**C**). Arrows indicate cells positive for PAX7 (green, **A**), and MYOGENIN (green, **B**). Confocal microscope image showing PAX7 positive cells (green, **C**). (**D**) Percentage of satellite cells (PAX7 positive cells) as quantified in several spheres from three independent biopsies. The blue line represents the median value of the data. (**E**–**J**) Serial confocal microscope images of d15-cultured spheres analysed by immunofluorescence for the detection of endothelial cell marker CD31 (**E**–**G**) and mesenchymal cell marker CD90 (**H**–**J**). Nuclei were counterstained with Hoechst 33258 (blue). Scale bars, 100 μm (**A**,**B**) and 50 μm (**C**,**E**–**J**).

To demonstrate the presence of myogenic cells in these cultures, myogenic markers PAX7 and MYOGENIN were detected by immunofluorescence in d7 myosphere cultures, showing discrete numbers of positive cells in the myospheres (Fig. 5A,B, arrows). Satellite (PAX7+) cells were quantified at day 15 of suspension culture (Fig. 5C). A median proportion of 16.2% PAX7+ cells (Fig. 5D) indicated that the sphere culture differentially enriched satellite cells over other cell types present at day 0, as previously observed in mouse dermosphere cultures^50^. To determine if endothelial (CD31+) cells and mesenchymal (CD90+) cells were also present in the cremaster-derived myosphere cultures, the presence of cells positive for these markers was analysed by immunofluorescence and confocal microscopy. Both CD31+ and CD90+ cells were easily detected, although quantification was difficult due to overlapping of the signal with numerous nuclei in the confocal sections (Fig. 5E–J). Interestingly, CD31+ cells seemed to form tube-like structures, reminiscent of angiotubes (Fig. 5E–G, arrows).

**Figure 6:**
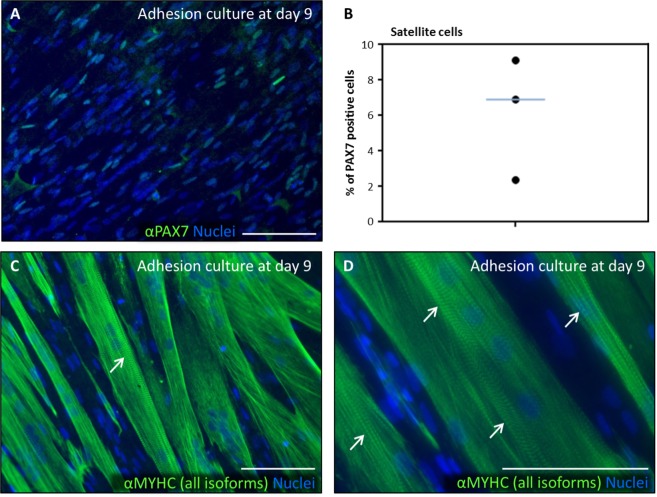
Expression of myogenic proteins during *in vitro* differentiation of human cremaster muscle-derived myogenic precursor cells. Immunofluorescence analysis of myogenic protein expression in d9-adhesion culture showed PAX7 positive cells (green, **A**), and MYHC (all isoforms) positive myotubes (green, **C**–**D**). Arrows indicate sarcomeric striations. (**C**) Percentage of PAX7 positive nuclei as quantified in three independent biopsies. The blue line represents the median value of the data. Nuclei were counterstained with Hoechst 33258 (blue). Scale bars, 100 μm.

**Figure 7:**
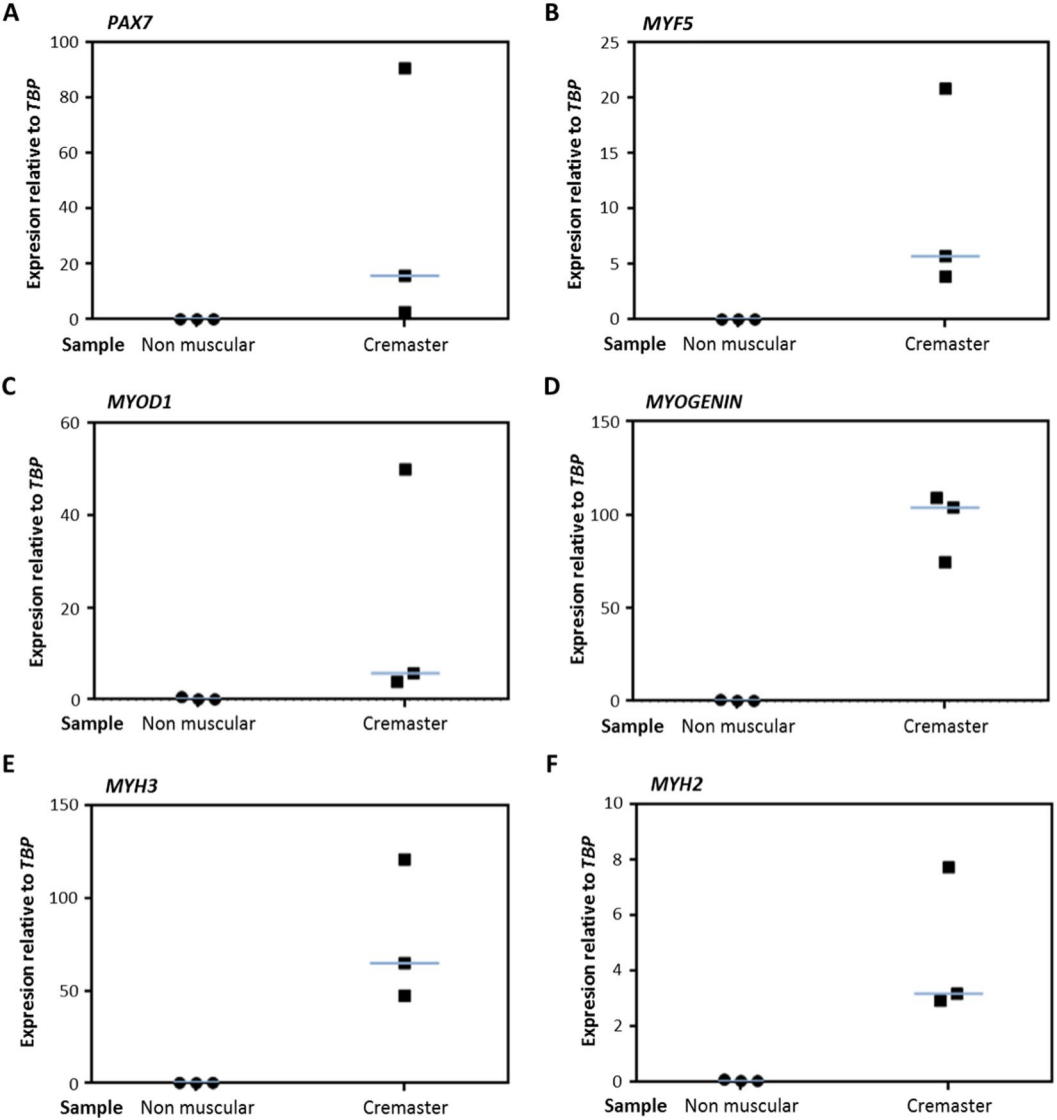
Expression of myogenic genes during *in vitro* differentiation of human cremaster muscle-derived myogenic precursor cells by qRT-PCR. At d9-differentiation cultures, the expression of myogenic genes *PAX7* (**A**), *MYF5* (**B**), *MYOD1* (**C**), *MYOGENIN* (**D**), *MYH3* (**E**) and *MYH2* (**F**) was quantified by qRT-PCR. The expression values from three independent cremaster muscle biopsies and noncremasteric biopsies are shown relative to endogenous control mRNA *TBP*. The blue lines represent the median values of the data.

After 9 days in differentiation culture (Fig. 6), single cells expressing PAX7 were still detected (Fig. 6A), with a median proportion of 6.9% PAX7+ cells (Fig. 6B). They were often observed in positions adjacent to MYHC+ multinucleated myotubes, which presented characteristic sarcomeric striations (Fig. 6C,D, arrows). The expression of myogenic genes was confirmed by qRT-PCR after 9 days in differentiation culture (Fig. 7). As negative control, patient biopsies that were taken from non-muscle tissue adjacent to the cremaster were used (Table 1). The cultures that were derived from cremaster muscle, but never those derived from non-muscle tissue, expressed variable but consistent amounts of the myogenic genes *PAX7*, *MYF5*, *MYOD1*, *MYOGENIN*, *MYH3* and *MYH2* (Fig. 7A–F). These results confirmed that cells of myogenic commitment were maintained by myosphere culture and were differentiated to myotubes in adhesion culture.

### *In vivo* regenerative potential of human cremaster muscle-derived cells in a mouse model of muscular damage

**Figure 8:**
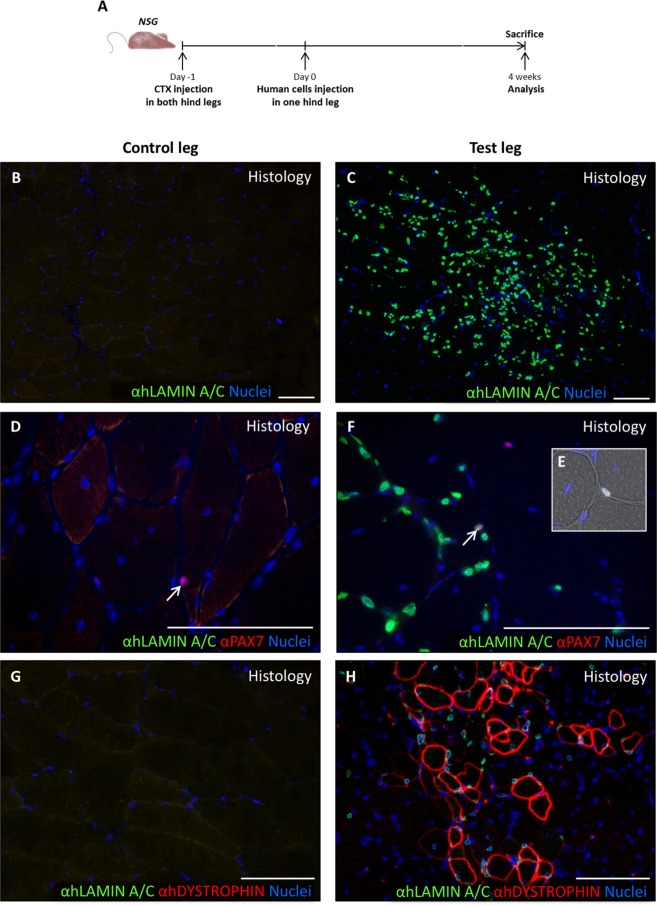
*In vivo* differentiation of human cremaster muscle-derived myogenic precursor cells. (**A**) Outline of the experimental design. (**B**–**H**) Immunofluorescence of histological sections from the control TA group (**B**,**D**,**G**) and the experimental TA group (**C**,**F**,**H**), respectively. (**B**,**C**) Histological analysis of the grafted cell survival through human LAMIN A/C detection by immunofluorescence (green). (**D**–**F**) Detection of human LAMIN A/C (green) and PAX7 (red) expressing satellite cells localized in their niche by immunofluorescence (arrows, **E**). (**G**,**H**) Detection of human LAMIN A/C (green) and human DYSTROPHIN (red) positive fibres. Nuclei were counterstained with Hoechst 33258 (blue). Scale bars, 100 μm.

**Figure 9:**
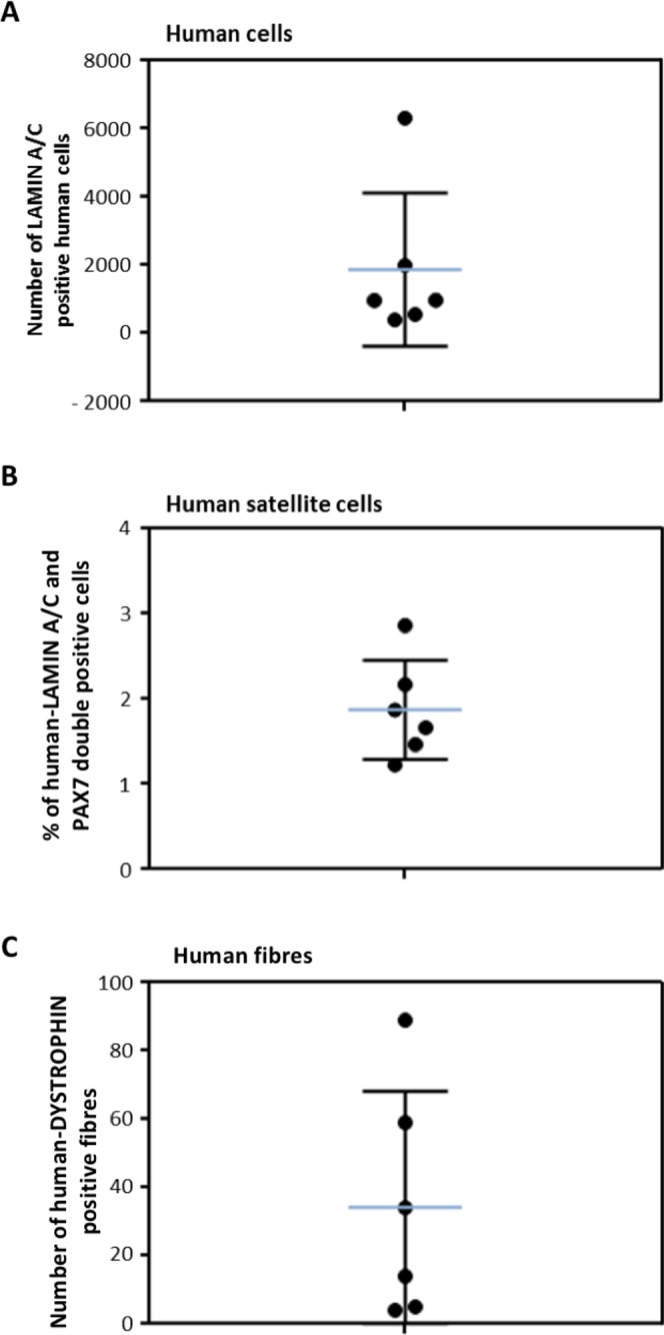
*In vivo* regenerative capacity of human cremaster muscle-derived myogenic precursor cells. (**A**) Quantification of the human LAMIN A/C positive nuclei detected in each mouse (N = 6). (**B**) Percentage of satellite cells (PAX7 positive cells) in relation to the total human nuclei detected in each mouse. (**C**) Quantification of the number of human DYSTROPHIN+ fibres per mouse. Median and standard deviation of the data are represented with blue and black lines, respectively.

To evaluate the potential use of human cremaster muscle-derived cells in future cell-based therapy trials, we analysed their regenerative potential in a proof-of-concept preclinical assay of muscle regeneration (Fig. 8A)^52,53^. Four weeks after cell injection, TA muscles were extracted and analysed by immunofluorescence. In the experimental TA muscle group (N = 6), a variable number of human cells was detected by their reactivity for the highly specific anti-human LAMIN A/C antibody, which was absent in the control leg (Fig. 8B,C). Of the LAMIN A/C+ cells, a relatively small percentage co-expressed the satellite cell marker PAX7 (Fig. 8D–F, arrows). The number of muscle fibres of human origin, as determined by the expression of human DYSTROPHIN by immunofluorescence (Fig. 8G,H), correlated quite well to the number of human cells detected per mice. Quantification of these human-specific markers (Fig. 9A,B) showed an average number of 1864 ± 2247 LAMIN A/C+ cells per section, of which 1.8 ± 0.6% were also PAX7+. The number of fibres of human origin (detected as hDYSTROPHIN+) was highly variable, 34.2 ± 34.0 fibres per section (Fig. 9C). These results suggested that human cremaster muscle-derived stem cells survived to the transplants and contributed to muscle regeneration in response to cardiotoxin damage.

## Discussion

Cell-based therapeutic approaches for muscle regeneration may be applicable in congenital myopathies^54^, muscular dystrophies and other neuromuscular diseases^55^, cardiac dysfunction^56^, volumetric muscle mass loss, cachexia and sarcopenia^57^. However, several of these pathologies affect relevant volumes of muscle tissue and this fact compromises the feasibility of cell-based approaches. For this reason, smaller muscle groups such as those affected in oculopharyngeal or in facioscapulohumeral muscular dystrophies may be approached with greater chances of success^35,58^. Anal and urinary sphincter deficiencies may also be excellent targets for these therapies^59,60^ since the muscle volume to be regenerated is small and the defect accessible via minimally invasive surgical approaches. For any of these pathologies it will be instrumental that adequate animal models are developed due to the inherent variability seen in the clinical setting^61^.

Despite the fact that the *Panniculus carnosus* muscle is vestigial in humans^43^, we were interested in testing if those evolutionary remnants still available in human beings would be of use to isolate muscle satellite cells of possible use in cell therapy, as we had previously done in the mouse^50^. The cremaster muscle was selected because it can be biopsied through a small inguinal incision which is routinely performed in several common urogenital surgeries in males. It is a predominantly slow twitch muscle and thus the number of satellite cells would be expected to be higher, although this seems to be dependent on muscle loading^62^. In this work, we found a satellite cell proportion *in situ* that seems to be below what has been established for other muscle groups^63^. The percentage of centrally nucleated (regenerative) fibres was on a similar range to other muscle groups^64,65^.

Nevertheless, we have also shown that cremasteric myogenic precursor cells can be maintained and expanded *in vitro* reaching sizable numbers (about 15% of the myosphere cells), and that the expanded cells retain muscle regenerative capacities *in vivo*. The main limitations of this initial study are: (i) the low number of samples analysed; (ii) the fact that this muscle can only be found in males, leaving aside half of the adult population; and (iii) that we characterized samples from patients and not from healthy individuals. For instance, higher grade varicoceles might be associated with denervation of cremaster muscle, causing small group atrophy^66^. These issues will be addressed by future investigations.

To apply cremaster-derived myogenic precursor cells in muscle pathology, a number of obstacles must be overcome: first, it must be studied if this muscle is affected by the relevant pathology at study, and second, an analogous muscle group should be found in adult women. A possible candidate could be the round ligament of the uterus, which is also composed of striated and smooth muscle fibres and that extends from uterus to deep inguinal ring, sometimes inserting itself into adipose tissue and skin of labia majora.

Importantly, the human cremaster muscle-derived myospheres were also shown to present mesenchymal (CD90+) and endothelial (CD31+) cells, and we postulate that, most likely, they will contain Schwann cells as well^67^. Human bone marrow-derived mesenspheres are CD90+, and contain bone marrow-derived stem cells which preserve an immature phenotype^68^. Tissue-resident CD31+ endothelial precursor cells have previously been isolated from mouse muscle^69^. Endothelial cells in co-culture spheres may self-assemble and form reticulated structures, as demonstrated here and previously seen in other systems^70,71^. The presence of both of these cell types might contribute to an improved vascularization of the affected area, as demonstrated in bone defects^72^. Finally, if Schwann cells and other peripheral nerve-derived cells were also present in these spheres, they might support reinnervation and regeneration of the degenerated tissue^73^. We would like to propose that, due to their origin and location, the mix of precursor cells present in the myospheres may be a good candidate to be used in cell therapy approaches of stress urinary incontinence after radical prostatectomy. Ideally, the isolation protocol should be adjusted so that precursor cell extraction and treatment may be done in the course of a single intervention^74,75^. Similarly, CD56+ myoblasts from pyramidal muscle have been obtained from radical prostatectomy patients^76^ and myoblasts derived from rectus muscles in patients undergoing open abdominal surgery^77^. Our approach would provide a more comprehensive pro-regenerative cellular mix.

## Conclusions

Human cremaster is a predominantly low twitch muscle with relatively few satellite cells and some regenerative fibres *in situ*. Cremaster-derived myogenic precursor cells can be isolated and expanded as myospheres, and the expanded cells retain muscle regenerative capacities *in vivo*. We propose that these precursors are a good candidate for cell therapy approaches of skeletal muscle. Due to their location and developmental origin, they might be best suited for the treatment of stress urinary incontinence after radical prostatectomy.

## Methods

### Aim, design and setting of the study

This study aimed to characterize *in vitro* and *in vivo* human cremaster muscle stem cells isolated from male donor biopsies (N = 19) and propagated *in vitro* in the form of myospheres.

### Ethics approval and consent to participate

The protocol to obtain cremaster and non-muscle tissue biopsies from patients after signature of the informed consents followed all relevant legal and ethical regulations, and was approved by the Ethics and the Scientific Committees of the HUVV (CEUMA No. 79-2015-A). Animal experiments were carried out following all relevant legal and ethical regulations, and according to protocols approved by the Biodonostia Animal Care Committee (CEEA16/008).

### Human cremaster muscle biopsies

Upon informed consent signature from male patients undergoing inguinal hernia (N = 10), hydrocele (N = 4), varicocele (N = 4), or spermatic cord cyst (N = 1) surgeries at the Virgen de la Victoria University Hospital (HUVV), a 1–3 cm^2^ cremaster muscle biopsy (or adjacent non-muscle tissue for negative controls) was collected during regular surgery procedure without adding any morbidity to the process. Biopsy samples were managed by the HUVV-IBIMA Biobank. Men with uro-oncologic disease or other diseases that could affect the scrotal area were not included as donors. The characteristics of biopsy donors are described in Table 1.

### Histology and immunohistochemistry

Deidentified human cremaster biopsies from surgery room (processed within 30 min of the procedure) were embedded in OCT medium (Q-Path, VWR), frozen by immersion in isopentane previously cooled in liquid nitrogen and stored at −80 °C until usage. Cryostat sections (7–10 μm) were stained with haematoxylin and eosin (H&E; Panreac), and mounted with Shandon Consul-Mount mounting media (Fisher Scientific) according to standard procedures. For immunohistochemistry, biopsy sections from OCT blocks were dried; and fixed in 4% Paraformaldehyde aqueous solution (Electron Microscopy Sciences) 10 min at room temperature (RT) or directly blocked with 5% bovine serum albumin (BSA) in PBS for 1 h at RT; and incubated overnight at 4 °C with the primary antibody in 1% BSA solution. Slides were rinsed in PBS containing 0.025% Triton X-100 (Amresco) and incubated for at least 1 h with the appropriate secondary antibody in the same solution used for blocking. The slides were rinsed with PBS containing 0.025% Triton X-100. Nuclei were stained with 10 μg/ml Hoechst solution (Santa Cruz, SC-394039) for 2 min and slides were mounted with Fluoro-Gel mounting media (Electron Microscopy Sciences, Cat #17985–10). Sections were imaged with a Nikon Eclipse 80i fluorescence microscope.

### Isolation, proliferation, and striated muscle differentiation of muscle precursor cells

Deidentified human cremaster biopsies from surgery room (processed within 30 min of the procedure) were transported immersed on HBSS (ThermoFisher, 14170-088) with 1% Fungizone and 1% Penicillin on ice. Biopsies were mechanically dissociated and digested with a collagenase type IA solution (Sigma-Aldrich, 650 CDU/mg, 2 mg/ml) for 1–2 h at 37 °C under gentle shaking. Collagenase was inactivated with culture media and the resulted digested tissue was filtered through 40-μm Nylon Cell Strainers (Corning). Collagenase was removed by centrifugation at 1500 rpm for 5 min at RT.

The cellular pellet containing muscle-derived cells was resuspended and cultured in suspension as described^50^, to promote myosphere expansion in proliferation medium [Neurobasal A (ThermoFisher) with 2% 50X B27 supplement (ThermoFisher), 1% 200 mM L-glutamine [Sigma-Aldrich], and 1% penicillin/streptomycin (100×)], supplemented every two days with 2% low serum growth supplement (1×, ThermoFisher), 40 ng/mL epidermal growth factor (R&D Systems), and 80 ng/mL basic fibroblast growth factor (FGF2; R&D Systems). Differentiation of myospheres was performed in adherent culture as described^51^. First, extracellular matrix-coated glass coverslips were prepared by incubating a solution of Cultrex basement membrane extract (2.77 mg/mL; Trevigen), Netrin-4 (0.83 μg/mL), Netrin-G1a (0.83 μg/mL), and low molecular weight hyaluronan (2.5 mg/mL; R&D Systems) in phosphate-buffered saline (PBS; pH 7.4). For muscle induction, primary myospheres were gently disaggregated with a 0.25% trypsin-EDTA solution (Sigma-Aldrich) and resuspended in proliferation medium without added growth factors plus 10% fetal bovine serum (ATCC), before plating onto coated coverslips at a density of 79,000 cells/cm^2^. Every 2 days, half of the medium was replaced with a fresh medium.

### Immunofluorescence of myogenic markers in myospheres

Immunofluorescence was performed in suspended sphere cultures. For suspended spheres, 1 ml of suspended culture was collected and centrifuged at 1500 rpm for 5′ at RT. The cell pellet was washed with 1X PBS and fixed in 4% Paraformaldehyde aqueous solution (Electron Microscopy Sciences) for 10 min at RT in a rotating station. Spheres were washed in PBS, permeabilized for 1 h in 0.3% Triton X-100 (Amresco) in PBS (PBS-T) and 5% normal donkey serum (Santa Cruz, SC-2044) at RT in the rotation station. Spheres were incubated with the appropriate primary antibody diluted in PBS-T overnight at 4 °C, rotating. Next, cells were 1X PBS-washed 3 times (5 min each) and incubated with the appropriate secondary antibody diluted in PBS-T for 1 h at RT in darkness and rotating. Prior to mounting in Fluoro-Gel, spheres were incubated with 10 μg/ml Hoechst (Santa Cruz, SC-394039) for 2 min rotating at RT and in darkness and washed with 1X PBS. Images were obtained by using a Nikon Eclipse 80i microscope coupled to a Nikon Digital Sight camera or a Zeiss LSM800 Confocal Laser Scanning Microscope. Confocal z-stack images were taken with the 25x objective (1.96 μm between slides). Images were processed using ZEN blue software and ImageJ software.

### Gene expression in differentiated cultures

Total RNA was extracted from differentiated cultures from cremaster biopsies and non-cremasteric biopsies by miRNeasy Mini Kit (Qiagen) and converted into complementary DNA with the High-Capacity cDNA Reverse Transcription Kit (Applied Biosystems). Each cDNA sample was amplified in triplicates for the real-time quantitative PCR (qRT-PCR) analysis which was carried out using Taqman gene expression assays in the 7900 HT Fast Real-Time PCR System (Applied Biosystems). The cycling conditions were 95 °C/10 min followed by 40 cycles at 95 °C/15 s, 60 °C/1 min in a reaction mixture that contained 1x Taqman Universal PCR Master Mix and 1x Assay Mix in a final volume of 20 μl. The expression of the genes was represented relative to the housekeep gene *Tbp* expression.

### Intramuscular cell transplantation

Animal experiments were carried out following the experimental design of Darabi and colleagues^52,53^. One day before cell transplantation, 10-week old immunocompromised *NSG* mice (NOD.Cg-PrKdc^sid^Il2rg^tm1Wjl^/SzJ, JAX 005557) (N = 6) were anesthetized with isoflurane and *Tibialis anterior* (TA) muscle damage was induced in both rear limbs through injection of 7.5 μL of 100 μM cardiotoxin (CTX, Latoxan) by using a 26 s gauge Hamilton syringe (bevelled tip). 24 h later, cells were injected (235,000 cells in 15 μl of PBS pH 7.2) into right TA muscles, whereas the left TA muscles received the same volume of PBS (negative controls). Before transplantation, human cremaster-derived precursor cells had been cultured as myospheres for 7 days, dissociated with 0.25% Trypsin-EDTA, filtered through 70 μm Nylon Cell Strainers (Corning) and resuspended in 1X PBS. Animals were caged by groups with *ad libitum* access to food and water and they were monitored for 4 weeks until sacrifice. Animals were sacrificed with CO_2_ and engrafted muscles were removed and processed for histological analysis. Seven-micrometer serial transverse cryosections were cut at intervals of 100 μm throughout the entire muscles.

### Antibodies list

Primary antibodies used were anti-CD31 (CD31) (M0823; 1:30; Dako); anti-CD90 (CD90) (ab133350; 1:200; Abcam); anti-hDystrophin (DYSTROPHIN) (NCL-DYS3; 1:20; Leica); anti-hLamin A/C (LAMIN A/C) (ab108595; 1:200; Abcam); anti-Laminin (LAMININ) (L9393; 1:200; Sigma); anti-MYOD (MyoD1) (SC-377460; 1:50; Santa Cruz); anti-myosin heavy chain all fibers (MYHC) (A4-1025-c; 1:200; DSHB); anti-myosin heavy chain embryonic (MYH3) (F1.652; 1:50; DSHB); anti-myosin heavy chain fast fibers (MYHC II) (A4.74; 1:200; DSHB); anti-myosin heavy chain slow fibers (MYHC I) (A4.840; 1:200 M; DSHB); and anti-PAX7 (Pax7) (Pax7-c; 1:50; DSHB). Secondary antibodies used were donkey anti-mouse Alexa Fluor 488 (A21202; 1:500; Thermo Fisher), donkey anti-mouse Alexa Fluor 488 (A21042; 1:500; Thermo Fisher), donkey anti-mouse Alexa Fluor 555 (A31570; 1:500; Thermo Fisher), donkey anti-rabbit Alexa Fluor 488 (A21206; 1:500; Thermo Fisher), and donkey anti-rabbit Alexa Fluor 555 (A31572; 1:500; Thermo Fisher).

## Data Availability

All data generated or analysed during this study are included in this published article.
